# Dietary Management in Pediatric Patients with Crohn’s Disease

**DOI:** 10.3390/nu13051611

**Published:** 2021-05-11

**Authors:** Luca Scarallo, Paolo Lionetti

**Affiliations:** 1 Gastroenterology and Nutrition Unit, Meyer Children’s Hospital, 50139 Florence, Italy; luca.scarallo@gmail.com; 2 Department NEUROFARBA, University of Florence, 50139 Florence, Italy

**Keywords:** Crohn’s disease, children, nutrition, dietary management

## Abstract

It has been widely endorsed that a multifactorial etiology, including interaction between genetic and environmental factors, can contribute to Crohn’s Disease (CD) pathogenesis. More specifically, diet has proven to be able to shape gut microbiota composition and thus is suspected to play a significant role in inflammatory bowel disease (IBD) pathogenesis. Moreover, poor nutritional status and growth retardation, arising from several factors such as reduced dietary intake or nutrient leakage from the gastrointestinal tract, represent the hallmarks of pediatric CD. For these reasons, multiple research lines have recently focused on the utilization of dietary therapies for the management of CD, aiming to target concurrently mucosal inflammation, intestinal dysbiosis and optimization of nutritional status. The forerunner of such interventions is represented by exclusive enteral nutrition (EEN), a robustly supported nutritional therapy; however, it is burdened by monotony and low tolerance in the long term. Novel dietary interventions, such as Crohn’s Disease Exclusion Diet or Crohn’s Disease treatment with eating, have shown their efficacy in the induction of remission in pediatric patients with CD. The aim of the present narrative review is to provide a synopsis of the available nutritional strategies in the management of pediatric CD and to discuss their application in the dietary management of these patients.

## 1. Introduction

Crohn’s Disease (CD) is a chronic idiopathic inflammatory affection involving the gastrointestinal (GI) tract. CD is a lifelong, currently incurable condition, whose natural history is characterized by alternating quiescent periods and active flares of inflammation, progressing to bowel damage and subsequent considerable morbidity [1,2]. To date, approximately 10% of CD cases are diagnosed before the patient’s 17th birthday, with a progressive acceleration in incidence in the last few decades [3,4]. When arising during childhood or adolescence, CD typically presents with a more extensive/panenteric phenotype [5,6]. In addition, as the disease occurs in a period of important developmental milestones, such as growth and puberty, children and adolescents are in a particularly vulnerable situation and management strategies need to deal with these peculiarities [7,8]. The exact pathophysiology of CD remains undetermined. It has been widely endorsed that multifactorial etiology, including an interaction between genetic and environmental factors, can contribute to CD pathogenesis [9]. Advances in DNA sequencing have led to the identification of more than 200 risk loci [10], which are estimated to explain only 25% of the inheritability of inflammatory bowel disease (IBD) [11,12]. The recent progress in basic and clinical science has changed our appraisal of the role of non-genetic factors in IBD susceptibility [13]. The dramatic rise in the incidence of IBD in newly industrialized countries shifting to Western dietary habits [14] represents one of the foremost clues to the relevant influence of environmental factors, including diet, on IBD pathogenesis. Dietary components have proven to be able to shape gut microbiota composition [15,16] and intestinal wall permeability [17], altering the interaction between host and intestinal microbiota, the latter being addressed as a master regulator of metabolism and immune response [13,18,19]. More specifically, high animal or dairy fat, animal protein, wheat, emulsifiers and thickeners appear to be strongly associated with intestinal inflammation in animal models [13]. Historically, growth failure at disease presentation represents one of the hallmarks of pediatric IBD, especially in CD, where it has also been associated with underweight and malnutrition [20]. Growth failure and malnutrition in IBD develop from a combination among low caloric input owing to decreased food intake (because of abdominal pain or restricted diets), malabsorption, increased basal metabolism and chronic inflammatory condition [21]. Undernutrition and nutritional deficiencies are associated with poorer clinical outcomes, such as higher infection rates or in-hospital length of stay, and with higher postoperative complications [22]. Despite being acknowledged as a multifactorial disease, where genetics, the immune system and the environment interplay, leading to disease onset and maintenance, treatment of CD remains focused on immune suppression (such as corticosteroids, methotrexates, thiopurines and biologic agents such anti-tumor necrosis factor alpha (TNFα)) [23]. Furthermore, notwithstanding the expansion of medical treatments, CD is still associated with non-negligible morbidity, mainly owing to its progression to complicated disease [24]. Moreover, the use of immunosuppressant agents and biologic drugs bears with it an increased risk of serious infections and malignancies [25,26]. In turn, corticosteroid use in pediatric patients affected by CD is associated with growth impairment and bone maturation delay [27,28]. Lastly, the achievement of consistent rates of deep outcomes (such as mucosal healing), along with sustained maintenance of remission, are still unfulfilled objectives [29,30]. Multiple studies in children with CD have demonstrated the efficacy in the induction of remission, the excellent safety profile and the nutritional benefits of exclusive enteral nutrition (EEN) [31,32]. However, EEN has tolerability issues, limiting compliance and widespread clinical application [33]. From this perspective, multiple research lines have investigated the efficacy of novel dietary therapeutic strategies designed to allow access to food excluding potential harmful substances for intestinal wall integrity and microbiome [34,35]. Such dietary strategies may enable the simultaneous targeting of the induction and maintenance of remission, allowing medication sparing, with the modulation of the gut microenvironment and the correction of possible macro- or micronutrient deficiencies and optimization of nutritional status. With this narrative review, we aimed to provide a synopsis of nutritional assessment of children with CD, the available nutritional strategies and their application in the dietary management of these patients.

## 2. Materials and Methods

Aiming to gather a comprehensive overview of existing dietary strategies for the management of pediatric CD, we performed an extensive literature search in Medline (PubMed, inception to January 2021), using “Crohn’s disease”, “children”, “pediatric”, “diet”, “micronutrients”, “macronutrients”, “deficiency”, “nutrition”, “nutritional”, “management”, “mechanism” and “malnutrition” as keywords. Studies not in English were not included. There were no other specific inclusion or exclusion criteria for this narrative review.

## 3. Mechanisms and Clinical Implications of Undernutrition in Children with CD

**Figure 1:**
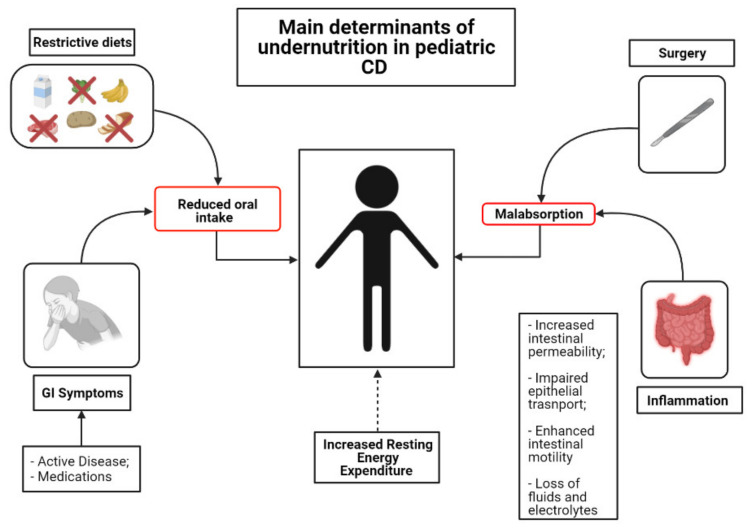
Mechanisms of undernutrition in children with CD.

The inflammatory involvement in CD may extend throughout the length of the small bowel, thus impairing the absorption and processing of nutrients [36]. However, several other factors contribute to development of inadequate nutritional states in IBD [37]. One of the foremost determinants of malnutrition in IBD is reduced oral intake of food. Active disease often leads to reduced appetite due to abdominal symptom onset (abdominal pain, diarrhea, vomiting and nausea) [38]. Moreover, inflammation itself (i.e., via TNF-α and IL-6) can cause a reduction in appetite via catabolic effects and hypothalamic weight regulation [39]. In addition, some of the most commonly prescribed medications can induce nausea, vomiting and/or anorexia [40]. Lastly, some patients and/or their parents believe that certain foods may worsen, or even elicit, their symptoms. Therefore, they are inclined to modify their diet, excluding putative noxious triggers, in order to control their disease. According to a recent European survey, the most commonly charged foods are grains (29%), milk (28%), vegetables (18%) and fruits (11%) [41]. This behavior may have detrimental effects on nutritional status [42]. The intestinal epithelium can be easily disrupted during gut inflammation [43]. Impaired epithelial transport and loss of mucosal integrity are tightly associated with malabsorption. Indeed, deterioration of epithelial function leads to alterations of ionic transport, which consequently cause loss of fluids and electrolytes [38]. Furthermore, inflammation of the intestinal mucosa results in chronic leakage of blood and proteins [38]. Surgery is also associated with impairment of macro- and micronutrient absorption [38]. Bowel resections can cause accelerated intestinal transit and diarrhea, thus reducing the contact time of the luminal contents with the mucosal surface. Lastly, conflicting evidence exists regarding Resting Energy Expenditure (REE). REE represents the energy needs for an individual in resting condition [44]. Increased REE is thought to contribute to augmented caloric requirements in patients with active IBD. However, the latter issue remains controversial, as some studies have documented a positive correlation between REE and disease activity [45], whereas some others have not [46] (Figure 1).

Linear growth impairment may be the first presenting symptom in up to 46% of children and adolescents affected by CD [47,48]. The prevalence of undernutrition and growth impairment has substantially decreased throughout the last few decades among patients with IBD [47,49,50]. However, irrespective of treatment modality, impaired linear growth and underweight still affect a significant proportion of pediatric patients with CD [51]. Underweight at diagnosis is associated with poorer disease outcomes [21,52]. In a retrospective longitudinal study, Yerushalmy-Feler reported that BMI in the lower quartile at diagnosis was associated with disease flares (HR: 3.212, *p* = 0.016) and with the need for anti-TNF- α (HR: 4.489, *p* = 0.021) in a cohort of children with IBD [52]. In a subsequent study from the same group, BMI in the lower quartile at 6, 12 and 18 months from diagnosis was associated with disease exacerbations (HR: 1.90, 1.98 and 2.43, respectively, *p* < 0.021) [21]. Children affected by CD have an altered body composition compared to healthy controls [53,54]. Thangarajah et al. [53] performed a systematic review aiming to define the alterations in non-bone tissue compartments in children with IBD. The results of the review showed that children affected by CD have a lower lean mass compared to healthy subjects [53]. In a recent prospective study, Ward et al. [55] enrolled 73 children with newly diagnosed CD to assess the impact of IBD on musculoskeletal health. The authors reported that total body lean mass (*z*-score −2.5, SD 1.1, *p* < 0.01) was low for age and gender. Furthermore, jumping mechanography demonstrated low muscle power [55]. Interestingly, children and adolescents with IBD show chronic deficits in lean body mass in spite of weight restoration and quiescent disease [56,57]. Persistence of lean mass deficiency negatively affects metabolic homeostasis, physical activity and bone mass accrual along with bone architecture and it is known to increase the risk of infections [58,59]. Moreover, lean mass deficit is known to affect negatively also some of the specific disease-related outcomes in IBD. In a retrospective study including 68 patients affected by IBD, Holt et al. [60] reported that lower values of skeletal muscle areas at TNF-α initiation were associated with a shorter time to loss of response. Despite advances in medical therapies, non-negligible numbers of patients during their disease course require a surgical intervention [61]. Unfortunately, undernutrition is a frequent clinical feature of children and adolescents who are referred to surgery. A long disease course, along with persistently active mucosal inflammation and the side effects of the multiple lines of medications, contribute to malnourishment in such patients. Undernutrition can have detrimental effects on post-surgical course. In a retrospective study on 161 CD patients who underwent elective ileocecal resection, poor nutritional status was independently associated with increased risk of postoperative septic complications [62]. Recently, the clinical implications of diminished lean body mass have been investigated by Ryan et al. [63]. Despite the vast heterogeneity in the assessment of sarcopenia, in a systematic review, the authors demonstrated that lean body mass deficit can predict the need for surgical intervention in patients with IBD and it is further associated with higher rates of major postoperative complications [63].

## 4. Nutritional Assessment and Supplementation

The identification of patients at risk of undernutrition is the first fundamental step aimed to detect those patients in whom nutritional and dietary interventions may bring major benefits. Nutritional assessment is a global outcome that encompasses multiple facets such as anthropometry measurements, clinical examination and assessment of dietary intake along with appraisal of socioeconomic environment. According to a recent position paper published by the European Society of Gastroenterology Hepatology and Nutrition (ESPGHAN) [44], weight, height and BMI *z*-scores should be used for assessment of the nutritional status at each visit in children affected by IBD. Evaluation of linear growth is of the utmost importance in children with CD, as it reflects both disease course and treatment success, and should be performed through height Standard Deviation Score (SDS) and height velocity SDS for a period of 6 to 12 months [44]. Moreover, as CD can be associated with delayed puberty, pubertal development should be closely monitored [44]. Pubertal stage should be assessed regularly from diagnosis in children aged 10 years and older, and at least annually during follow-up visits until puberty is completed [44]. Beyond the appraisal of nutritional status and pubertal development, it is likewise paramount to assess dietary intake as part of the follow-up for pediatric patients with CD. Patients and/or their caregivers may tend to self-restrict their diets [64]. Inadequate food intake, with lower consumption of energy, fiber, carbohydrates and several micronutrients, has been consistently described both in active disease and in remission in children affected by CD [65]. According to the last ESPGHAN position paper on Nutrition in Pediatric IBD, a 3 to 5-day dietary record is recommended as the best-suited method to perform the quantitative evaluation of energy and nutrient intake [44] and it should be performed at least annually in older children and at least twice per year in younger IBD patients [44].

### 4.1. Macronutrients

Macronutrients include proteins, carbohydrates and lipids. Children and adolescents affected by CD seem to have similar nutritional requirements to their healthy peers [44]. However, data arising from the few studies who investigated protein requirements in children and adolescents with CD suggest that, in phases of active disease, protein demand may be increased [66,67]. Protein breakdown has been demonstrated to be reduced after surgical intestinal resection in children with active CD [45]. Further, one small study including 15 pediatric CD patients undergoing metabolic assessment immediately before and after the first infliximab infusion reported that TNF-α therapy reduced proteolysis and improved the protein balance in parenterally fed patients [68]. These findings suggest that protein intake may be increased by 25% during the active phases of disease in order to mitigate protein loss [44]. To date, no studies have demonstrated a different requirement of carbohydrates and fats in children affected by CD.

### 4.2. Micronutrients

#### 4.2.1. Iron

Iron deficiency (ID) and Iron Deficiency Anemia (IDA) are frequently encountered clinical entities in pediatric IBD [69,70]. Using the World Health Organization definition of anemia, its prevalence at diagnosis reaches 78% in pediatric IBD populations [71,72,73]. Moreover, anemia affects up to 42% of children with IBD one year after diagnosis [69,74] and it has been reported to be more frequent in pediatric populations than in adult ones both at diagnosis and at follow-up [74]. Anemia of pediatric IBD has a complex and multifactorial pathogenesis, but the most common etiology arises from a combination of IDA and anemia of chronic diseases (ACD) [70]. More specifically, IDA has been reported as the leading cause of anemia in children with IBD and with CD [75,76]. Some reports have shown that, despite achievement of clinical remission, a non-negligible rate of children with IBD are affected by anemia at one-year follow-up [77]. These findings suggest that anemia is a relevant comorbidity for children with CD and that it may not resolve unless specific therapeutic interventions. When dealing with a child affected by IBD and anemia, the first therapeutic approach should include providing a balanced and diversified diet with iron-rich foods along with enhancement of alimentary iron absorption (i.e., pairing non-heme iron sources with food rich in ascorbic acid or avoiding coupling with foods that may impair iron absorption). Once having provided adequate control of disease activity and optimized dietary iron intake, the next step in case of persistence of IDA is represented by iron supplementation [70]. The choice of whether to administer oral or parenteral iron supplementation is not always straightforward [70]. Oral supplementation formulations have the relative advantage of not requiring infusions and thus scheduled access to the hospital, of being easily available and less expensive compared to parenteral products. However, concerns exist regarding their well-documented gastrointestinal side effects [78] and the possibility of adding a symptom burden in addition to those of IBD. Indeed, the intolerance rate is a frequent finding, leading to discontinuation in up to 50% of patients [79]. Moreover, some authors have suggested that oral supplementation with iron formulations may negatively impact intestinal inflammation [80,81]. More recently, a novel oral iron formulation, ferric maltol, consisting of a single ferric ion (Fe^3+^) chelated with high affinity to three maltol molecules, has been demonstrated to be safe, effective and well-tolerated in patients with IDA and IBD who had reported poor tolerance to other oral ferrous preparations [82]. Oral formulations have also been associated with alterations of gut microbiota [83]. However, in a recent prospective, controlled, open-label trial, Rampton et al. [84] demonstrated that oral iron supplementation did not increase disease activity in adolescents and adults with IBD over a period of 6 weeks. Therefore, oral iron supplementation may be used in children and adolescents with milder grades of anemia and inactive disease [70]. Besides oral iron compounds, different intravenous formulations are available as therapeutic options for IDA in patients with IBD. Whereas, historically, high-molecular-weight intravenous iron compounds were burdened by a significant rate of side effects and therefore underused due to safety concerns, the introduction of low-molecular-weight formulations led to a significant decrease in adverse effects [85]. Various studies performed in children and adolescents with IBD reported a high efficacy of intravenous iron formulations with a relatively low rate of adverse events [86,87,88,89]. Mamula et al. [86] reported their retrospective experience with intravenous iron dextran infusions in 70 children affected by IBD. The authors observed a significant rise in Hgb levels, with an average increase of 2.9 g/dL. Hypersensitivity reactions were rare: only 9% of patients experienced infusion reactions, none of which was severe [86]. Further, Powers et al. [90] reported their retrospective experience of the administration of intravenous ferric carboxymaltose in children affected by IBD who responded poorly to oral iron formulations. Among the 72 patients included in the study, a median Hgb rise of 3.2 g/dL was reported, with only seven children (16%) reporting minor adverse infusion reactions [90]. Intravenous iron preparations are effective and well tolerated. Their administration should be envisaged in children with CD with moderate-to-severe forms of anemia and in those patients who are intolerant or unresponsive to oral iron compounds [70].

#### 4.2.2. Vitamin B12 and Folate

Vitamin B12 status can be assessed by measuring either serum B12 levels or, more accurately, by methylmalonic acid and homocysteine levels [91]. Ileal disease or resection may mediate vitamin B12 malabsorption, placing CD patients at risk of deficiency [92]. Other postulated mechanisms for vitamin B12 deficiency include small bowel bacterial overgrowth, reduced intake, increased physiologic requirements and protein losing enteropathy [92]. A systematic review including 3732 patients from 42 studies concluded that CD without ileal resection (or with a resection of less than 20 cm) did not increase the risk of B12 deficiency [92]. A recent systematic review analyzed eight studies that investigated vitamin B12 status in children with IBD. The authors observed an overall low rate of B12 deficiency in the population studied [93]. One study reported that ileal or ileocolonic resection increased the risk of abnormal serum vitamin B12 concentrations [94]. According to the latest ESPGHAN workgroup recommendations [44], vitamin B12 screening should be performed in all patients with a history of ileal or ileocolonic resection and in those patients with suspected vitamin B12 malabsorption. When depleted, patients should be treated with intra-muscular injections [44].

Folate (vitamin B9) levels can be measured in serum or, more accurately, in red blood cells (RBC) and also, indirectly, by measuring serum homocysteine levels [44]. Studies conducted in adult CD patients have reported a rate of folate deficiency ranging from 20% to 30% [94,95,96]. Pediatric data are limited. However, folate deficiency appears to be infrequent in children affected by CD [93]. According to ESPGHAN’s recommendations, children should receive additional folic acid supplementation (1 mg daily or 5 mg weekly) when being treated with methotrexate (MTX), as it acts by inhibiting folate’s cellular uptake [44].

#### 4.2.3. Calcium and Vitamin D

Restriction of milk and dairy products consumption is a common issue in patients affected by IBD and CD [97,98]. Moreover, chronic inflammation, corticosteroid use, suboptimal nutritional status, delayed puberty and a sedentary lifestyle all contribute to the genesis of a low bone mineral density (BMD) in children with IBD [99,100,101]. Bone mass accrual occurs predominantly during the phase of transition from childhood to adolescence. Any disruption of such a complex and delicate phenomenon may have detrimental long-term consequences. Sigurdsson et al. [102] performed a prospective longitudinal study including 74 children affected by IBD followed into young adulthood. They reported that male patients with childhood-onset IBD had a BMD lower than reference values [102]. Anat Guz-Mark et al. [103] also reported similar results. Among the 61 young adult patients with pediatric-onset IBD, the authors reported a median BMD *z*-score significantly lower than those of the healthy population (*p* < 0.001), with a high prevalence of both osteopenia (defined as BMD *z*-score ≤ −1 SD, 44.3%) and osteoporosis (defined as BMD *z*-score ≤ −2.5 SD, 8.2%). Vitamin D deficiency has been consistently reported across several pediatric CD populations [104,105]. The latest guidelines recommend weight-based supplementation of vitamin D when deficiency is present (25(OH) D concentrations below 50 nmol/L or 20 ng/mL) [44]. Several strategies for vitamin D repletion exist. Various regimens are used, ranging from 4000 IU daily to 50,000 IU weekly [106]. Recently, Lee et al. [107] have also demonstrated, in a prospective randomized study, the efficacy of a single high dose of vitamin D of 300,000 IU. When compared to administration of a weekly dose of 50,000 UI, the authors observed an equivalent rise in vitamin D in the two groups (53.6 ± 17.3 ng/mL vs. 54.6 ± 17.5 ng/mL) [106], significantly far from toxicity levels [107]. Despite its pivotal role in the regulation of mineral and bone homeostasis, vitamin D plays a key part in the modulation of both the innate and adaptive immune response [108]. Low serum vitamin D levels have been demonstrated to be associated with disease activity and with poorer clinical course in adult patients with IBD [109]. Recently, El Amrousy et al. [110] conducted a double-blinded, randomized clinical trial on 120 children and adolescents affected by IBD and hypovitaminosis D who were randomized to receive either oral vitamin D3 in a dose of 2000 IU/day or placebo for 6 months. The authors reported that vitamin D supplementation was significantly inversely associated with clinical activity scores (PCDAI: 13.6 ± 3.1 in treatment group vs. 27.5 ± 3.5 in placebo group; PUCAI: 11.1 ± 2.4 in treatment group vs. 21.8 ± 2.9 in placebo group; *p* = 0.001) and with serum and fecal inflammatory markers (C-reactive protein (CRP), fecal calprotectin (FC) and pro-inflammatory cytokines). A recent systematic review with meta-analysis of adult studies, including 970 patients with IBD from 12 randomized controlled trial (RCT) and 4 observational studies, demonstrated that supplementation of vitamin D significantly increased serum levels of 25(OH) vitamin D in both UC and CD patients. Lastly, the analysis revealed a reduction in clinical activity indices (Harvey-Bradshaw Index) and of inflammatory markers (high-sensitivity CRP) after supplementation with vitamin D [111].

### 4.3. Dietary Fiber

Dietary fiber describes a complex group of non-digestible components of cell walls [112]. Fibers can withstand the gastric acidity and are not metabolized by human gut cells. In the large bowel, dietary fiber serves as substrates for fermentation by the gut microbiota. Such a process produces byproducts, such as short-chain fatty acids (SCFAs) [113]. The main SCFAs (acetate, butyrate and propionate) have been demonstrated to positively affect the host in several ways, including offering energy supply to colonic epithelial cells and reducing the extent of inflammation in IBD [114]. Dietary fiber restriction has been associated with a higher consumption of colonic mucus by bacterial species, potentially contributing to inflammation [17]. Dietary fiber intake of children with IBD has been demonstrated to be suboptimal when compared to healthy controls [115] or recommended intake guidelines, regardless of whether the patient had active or inactive disease [116]. According to the recent recommendations from the Nutrition Cluster of the International Organization for the Study of Inflammatory Bowel Diseases, there is currently no evidence to support the restriction of dietary fiber in patients without intestinal strictures or obstructions [117].

## 5. Nutritional Therapies

Nutritional status represents one of the foremost determinants of both clinical and surgical outcomes for patients affected by CD [118]. From this perspective, the identification, prevention and correction of nutritional deficiencies can be considered a therapeutic intervention as crucial as the choice of adequate pharmacological strategies. Indeed, malnutrition and impaired linear growth can be suggestive of active disease and their restoration should be considered as a treatment goal [44].

### 5.1. EEN

EEN is a nutritional treatment strategy that provides the total amount of calories and nutrient requirements of patients through a proprietary liquid formula administered orally via an enteral tube. There are three main types of EEN currently available, namely elemental (amino-acid-based), semi-elemental (oligopeptide-based) and polymeric (whole-protein-based) formulas [119]. Elemental formulas are entirely antigen-free, and they are best-suited in cases of severe malabsorption, where there may be impairment of the gastrointestinal tract. Oligopeptide formulas are peptide-based feeds where the source of nitrogen is represented by peptides of four or five amino acids in length obtained from hydrolysis of whole proteins. Lastly, polymeric feeds contain whole proteins from milk, egg, soy or even meat [119]. Efficacy of EEN does not vary according to the type of feed, as it also has been recently confirmed by a Cochrane review of EEN for the induction of remission in CD that evaluated the different formulas composition and found no difference in efficacy or in side effects [120]. However, polymeric feeds are more palatable when consumed orally and they are more commonly used to improve adherence to treatment, helping to overcome one of the major limitations of EEN treatment [121]. EEN has no major medical side effects. The more commonly reported adverse effects by patients on EEN include diarrhea, nausea and vomiting. Moreover, in pediatric CD, in extremely selected cases with prolonged nutritional restriction, abrupt re-introduction of calories may be associated with refeeding syndrome [122,123].

#### 5.1.1. Mechanism of Action

**Figure 2:**
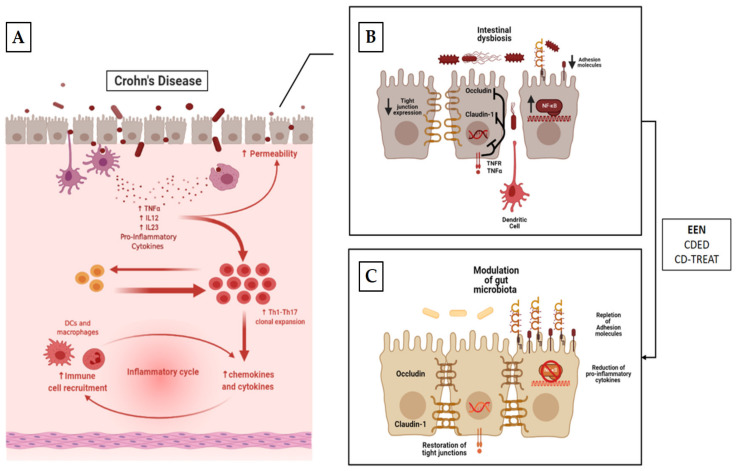
Mechanism of action of EEN, CDED and CD-TREAT. The panels (**A**,**B**) depict the potential pathogenic effects of diet on the development of CD. (i) Reduction in the expression of apical membrane adhesion molecules, which cause increased interactions between bacteria and epithelial membrane. (ii) Reduction in the synthesis and expression of lateral tight junction proteins, causing defects in gut barrier. As a consequence, there is an amplification of the interaction between gut bacteria and immune cells, causing stimulation of pro-inflammatory cytokine secretion and attracting and stimulating the proliferation of pro-inflammatory T-cells with the establishment of a pro-inflammatory milieu along with a self-empowered mechanism. Panel (**C**) describes the potential effects of nutritional interventions. (i) Modulation of gut microbiota. (ii) Enhancement of expression of apical adhesion molecules. (iii) Increased expression of tight junction with restoration of intestine permeability. (iv) Reduction in pro-inflammatory cytokines and, as a consequence, polarization of immune response towards a regulatory phenotype of T cells.

Impaired epithelial barrier function, alterations of normal gut microbiota composition and translocation of bacteria through the intestinal epithelium have all been described in IBD and thus in CD [13,124,125,126]. Despite the wide availability of data regarding EEN utilization for the induction of remission in CD, there is a considerable knowledge gap in our understanding of its exact mechanism of action. EEN has the ability to mitigate the aberrant immune response that characterizes CD and thus to relieve inflammation. This has been highlighted by several in vivo and in vitro studies [127,128,129]. EEN is thought to exert its anti-inflammatory properties through deactivation of the major intracellular signaling pathways NF-κB [129,130]. Two amino acids contained in the polymeric formula, arginine and lysine, have been addressed as responsible for this effect via interference with its kinase activity [131]. In an in vitro study, the polymeric formula has also been demonstrated to reduce the production of pro-inflammatory cytokines as IL-6, IL-8, IL-1b and IFN-γ by peripheral blood mononuclear cells isolated from children with active CD [132]. Besides the reduction of pro-inflammatory cytokines, EEN has also been shown to be able to enhance TGF-β1 [133], an immune-suppressive cytokine whose signaling pathway has been detected in patients with active CD’s intestine [134]. Consequently, some authors have postulated that enrichment of EEN formulations with TGF-β might boost their anti-inflammatory properties [135]. Further, EEN has been demonstrated to increase the expression of some of the adhesion molecules involved in the innate intestinal immune response. Repletion of such transmembrane proteins may prevent bacterial interaction with intestinal epithelial cells and thus their activation of dendritic cells, macrophages and the mucosal inflammation cascade [136,137]. EEN has also been shown to improve mucus integrity and gut permeability in children affected by CD. This effect is thought to be exerted by: (i) avoidance of dietary products that impair intestinal barrier [13]; (ii) reduction of pro-inflammatory cytokines that have been implicated in the reduction of permeability [138]; (iii) restoration of cell-to-cell adhesion molecules (such as occludin and claudin-1) [138] (Figure 2).

Pediatric patients with newly diagnosed CD display reduced microbial diversity when compared to healthy controls [139], with a relatively lower representation of Firmicutes as well as an expansion of Proteobacteria [140]. Moreover, the intestinal metabolic signature of patients with CD, including short-chain fatty acid (SCFA) reduction [141,142], amino acid abundance [142] and bile acid composition dysregulation [143], pronouncedly differs from those of healthy controls. Modulation of gut microbiota along with intestinal metabolic changes are among the likely mechanisms through which EEN exerts its functions [144,145]. Interestingly, some studies have demonstrated that EEN administration is associated with a reduction in the alpha-diversity of gut microflora, a parameter usually associated with microbiota “fitness” [146]. Additionally, a reduction in SCFAs such as butyrate, a presumed beneficial metabolite for the host, has been observed during successful EEN induction treatment [146]. However, it is worth noting that these findings are not so surprising when looking at the composition of EEN. More specifically, shortage of complex fermentable carbohydrates reduces SCFA substrates, whereas the limited number of EEN components, compared to a regular diet, may account for the reduction in microflora diversity [144]. Indeed, although fiber has been considered to exert a potential clinical benefit for patients with CD, the vast majority of EEN formulations do not contain any fiber [147]. Moreover, among the few EEN formulations containing fiber, the main ones were slightly different from the range of fibers included in standard diets. The latter observation, however, does not suggest that fiber is harmful, but rather that its removal from the diet does not worsen symptoms in patients with CD [147].

Some authors have hypothesized that that EEN may exert its effect by depleting harmful bacteria and thus allowing for subsequent re-colonization [148]. Lastly, a recent prospective multicenter cohort study including 43 newly diagnosed children with CD receiving EEN as induction therapy reported decreased microbiota diversity, a reduction in fecal amino acid concentration and a partial regularization of the microbial metabolism of bile acids [144]. Moreover, differences in microbiota and metabolome patterns were observed between responders and non-responders to EEN, postulating the possibility for future prediction of EEN response.

#### 5.1.2. EEN for Induction of Remission in CD

In the early 1970s, Voitk et al. [149] reported for the first time in an uncontrolled study the use of Enteral Nutrition (EN) as a management option for active IBD. Thirteen patients awaiting surgery were treated with an elemental diet for an average time of 22 days. The authors observed improved nutritional states and, unexpectedly, a clinical improvement, with some even avoiding the intended surgery. Subsequently, the first experiences with elemental diet have been reported also in pediatric populations [150,151], showing effectiveness of a 6-week course of elemental diet in restoring growth [150] and inducing remission [151]. Following the above-cited pathfinder studies, several other research lines, including several meta-analyses [120,152], demonstrated the efficacy of EEN for the induction of remission in patients, especially children with CD. According to this recent body of literature, EEN may induce remission in up to 86% of children with CD, along with a significant decrease in inflammatory biomarkers, such as C reactive protein (CRP), erythrocyte sedimentation rate (ESR) and FC [27,153,154]. Furthermore, in a multicenter prospective inception cohort of newly diagnosed mild-to-moderate CD, Cohen-Dolev et al. [27] showed that, in a propensity-matched score analysis, EEN was superior to corticosteroids for induction of remission (*p* = 0.05) and preservation of linear growth (*p* = 0.055). Efficacy of EEN in the induction of clinical remission in children with CD was further confirmed by Lee et al. [154] also in comparison to biological therapy. Ninety consecutive children with active CD were consequently enrolled in a prospective study, receiving EEN, Infliximab (IFX) or partial enteral nutrition (PEN) for the induction of remission. No differences in clinical response rates between EEN and biologic therapy were observed (88% vs. 84%). Even though symptom control is a relevant endpoint for patients with IBD, more recently, treatment strategies have evolved, moving towards targeting the resolution of mucosal inflammation [155]. Several studies demonstrated that EEN is also effective in inducing mucosal healing (MH) within 8–10 weeks of its initiation [156]. In a prospective, open-label study including 26 children with newly diagnosed CD who completed a 6-week EEN course, 15 (58%) had early endoscopic response, with 11 (42%) of them showing complete MH (Simplified Endoscopic Score for CD (SES−CD) = 0). The authors reported also complete transmural remission in 3 out of 14 (21%) patients with ileal CD [156]. Additionally, EEN has also outperformed corticosteroid treatment in MH achievement when the two strategies have been directly compared. In an open-label, randomized controlled trial enrolling 37 children with recently diagnosed active CD, the proportion of patients showing MH was significantly higher in the EEN group than in the corticosteroid group (74% vs. 33%, *p* < 0.05) [157]. Furthermore, achievement of complete MH after administration of EEN has been shown to predict also long-term sustained clinical remission [158]. In a prospective single-center study, Grover at al. [158] demonstrated that, among 54 children with mild-to-moderate CD completing a 6-week EEN induction course, achievement of complete MH (SES-CD = 0) was associated with higher sustained remission at 3 years compared with active endoscopic disease (8/16, (50%) vs. 1/19, (6%), *p* = 0.005). Conflicting data regarding the association between EEN efficacy and CD localization are available [31,159,160,161]. Previous studies suggested a lower efficacy of EEN in patients with isolated colonic disease compared to those with ileal involvement [160]. Subsequent experiences, however, found no differences between isolated colonic disease and ileal or ileocecal involvement [31,161]. In the absence of high-quality evidence, EEN is recommended as the first-line treatment irrespective of CD location [23,44]. Moreover, in cases of the second course of EEN, studies have shown good efficacy, with clinical remission rates ranging from 58% to 80%. Moreover, two small prospective studies demonstrated that intermittent administration of EEN over one year resulted in a reduction in corticosteroid need and with increased growth velocity [162,163]. These data confirm that if the compliance of the patients is maintained, EEN may be successfully reused during the subsequent course of the disease for future relapses [44] (Table 1).

#### 5.1.3. EEN for Preoperative Nutritional Optimization in Children with CD

As previously mentioned, surgery in children with CD is often performed when therapeutic inventory has been depleted, a setting where the onset of postoperative complications is likely to be substantial due to the burden of immunosuppression, hospitalization and malnutrition. Nutritional status is a key element for favorable surgical outcomes [164]. From this perspective, perioperative nutritional therapy has been addressed as a pivotal element when caring for patients with CD who require surgical interventions [118]. A recent systematic literature review performed by a consensus expert panel acknowledged the use of nutritional therapies prior to surgery as a perioperative optimization strategy, despite the paucity of available pediatric data [118]. Indeed, although EEN was first conceived as a preoperative nutritional support, there is a dearth of high-quality studies reporting its systematic use in a preoperative setting [165,166]. A recent systematic review explored preoperative nutritional conditioning of patients with CD [167]. Fourteen original studies were identified. Five out of the studies that included a control group [165,168,169,170,171], four of which utilized EEN and one parenteral nutrition (PN), reported improved post-surgical outcomes in patients receiving preoperative nutritional support. To date, the only experience reported in pediatric populations is represented by a small case series reported by Harris et al. [172], who assessed the use of EEN as a treatment prior to resection surgery for pediatric CD patients. The authors gathered data on 17 CD pediatric patients who underwent right hemicolectomy. Eight of them received a preoperative course of EEN for a median time of 4 weeks (IQR: 3.7–4.0 weeks). The median length of hospital stay after surgery was lower in the EEN cohort compared to the non-EEN one (4.5 days versus 6 days, *p* = 0.03). Moreover, the use of EEN was associated with a decreased rate of moderate or severe disease on resection pathology (5/8 (62.5%) versus 9/9 (100%); *p* = 0.04). No differences in serologic markers were observed, in contrast to previously published adult literature, arguably due to the small sample size. Replication of the results of this study, along with well-designed prospective studies, hopefully RCTs, are deemed to substantiate EEN’s role in the preoperative setting of pediatric CD.

#### 5.1.4. EEN’s Effects on Body Mass Composition in Children with CD

The benefits of EEN extend beyond its ability to induce remission and its capacity to improve anthropometric indices in patients with CD. Gerasimidis et al. [32] performed a prospective study enrolling 17 children with active CD who received EEN, aiming to assess its effects on changes on body composition [32,173,174]. The authors reported an improvement in lean body mass after EEN course (lean body mass *z*-score: −2.1 vs. −0.8 before and after treatment, respectively; *p* < 0.0001) without a significant increase in fat mass [32]. In the same study, also a significant improvement in circulating micronutrients was observed after EEN treatment [32]. A more recent prospective study performed by Strisciuglio et al. [174] reported that EEN was associated with an improvement in both fat free mass (assessed via bioimpedentiometric examination) and bone mineral density (assessed via dual-energy X-ray) in children with newly diagnosed CD. Moreover, the authors observed a significant increase in bone mineral density also at the 1-year follow-up visit, supporting the hypothesis that the reduction in inflammation combined with improvement in nutritional status might be persistent after one year, resulting in ameliorated bone composition and muscle mass. Lastly, a recent sub-study of a randomized clinical trial enrolling children with new-onset mild-to-moderate CD explored the effect of nutritional therapies on bone health. The authors evaluated bone formation and resorption at baseline, at week 12 and at week 24 via serum C-Propeptide of Type I Procollagen (CICP) and type I Collagen N-Telopeptide (NTX), respectively [175]. Bone mineral density was assessed by dual-energy X-ray. The authors reported a significant improvement in CICP, a sensitive marker of bone formation but not of bone mineral density, suggesting that bone improvement is a challenging and a slow process [175].

### 5.2. Partial Enteral Nutrition (PEN) in CD

PEN refers to the replacement of 35% to 50% of habitual food intake with EN feeds while continuing to eat an unrestricted or exclusion diet.

#### 5.2.1. PEN for the Induction of Remission in CD

The first RCT specifically designed to investigate the efficacy of PEN (provided as 50% of elemental formula associated with unrestricted diet) in the induction of remission in children and adolescents with active CD showed unsatisfactory clinical remission rates. Among the 50 children who were randomly assigned to receive either 50% (PEN) or 100% (EEN) of their energy requirement as elemental formula for six weeks, clinical remission rates were significantly lower in the PEN group compared to the EEN one (15% vs. 42%) [176]. These results suggest that PEN with access to a free diet is ineffective at relieving inflammation when compared with EEN or IFX, restating the principle of exclusivity. Following the results of this robustly designed, groundbreaking study, research interest in PEN as an alternative induction treatment for CD diminished. More recently, Gupta et al. [177] reported the efficacy of a nutritional treatment protocol involving the administration of 80% to 90% of caloric needs through an EN formula overnight and the remaining 10% to 20% of the daily energy intake from an unrestricted diet. Among 43 CD pediatric patients included in this retrospective study, induction of remission was achieved in 65% of them, within a mean treatment duration of 2 months. However, this nutritional protocol led to frequently reported adverse events (up to 65% of patients) mainly related the use of nocturnal EEN (abdominal distension, sleep disturbances, morning nausea and vomiting, etc.). Nevertheless, these results have not been replicated in more robustly designed studies. Therefore, PEN associated with an unrestricted diet is not currently recommended for the induction of remission of children and adolescents with CD [23].

#### 5.2.2. Maintenance of Enteral Nutrition (MEN) in CD

Long-term exclusive enteral nutrition and avoidance of a normal diet is an unfeasible therapeutic option for the maintenance of remission, mainly owing to compliance issues. Maintenance of enteral nutrition (MEN) refers to the use of EN formula, accounting for a percentage of the total caloric requirements, for the maintenance of remission in CD. In a retrospective historical cohort, Wilschanski et al. [178] first reported the effectiveness of supplementary administration of an elemental formula in a cohort of children and adolescents affected by CD. Among 47 pediatric CD patients who achieved remission after EEN induction, 28 of them continued nocturnal supplementary feeding via a nasogastric tube after resumption of an otherwise unrestricted daytime diet. This subset of patients showed a higher cumulative probability of maintaining clinical remission compared to the group of 19 patients who did not receive PEN (*p* = 0.005). A subsequent retrospective study showed that up to 50% of patients who received MEN as the only maintenance therapy after a successful course of EEN remained in remission at 1-year follow-up [179]. This response rate did not differ from those obtained with azathioprine (65%) or with the combination of MEN and azathioprine (67%) but was significantly higher than those achieved without maintenance therapy (15%). Conversely, subsequent studies were not able to confirm the association between MEN and prolonged time to relapse [180,181,182]. A retrospective study performed by Gavin et al. [181] did not observe a difference in remission length between patients who received MEN and those who remained on a habitual diet (77% vs. 78% 1-year relapse rates). Moreover, in a recent study, Logan et al [182] demonstrated that patients who used MEN during the early period of food reintroduction following a successful course of EEN had lower FC levels compared to those who did not consume MEN. Nonetheless, this effect appeared to be only short-lived as the use of MEN was not associated with prolongation of remission length. Beyond the sole clinical efficacy, the use of MEN has been associated with additional benefits, such as increased height [178] and weight [180] z-scores in children and adolescents. The main evidence to support the benefits of MEN to prolong remission following a successful course of EEN is supported by two RCTs performed in adult cohorts in Japan [183,184]. Furthermore, a recent meta-analysis investigated the effects of EN in combination with anti-TNF-alpha antibody treatment in adult patients affected by CD [185]. The authors demonstrated, via a systematic review of nine studies, that maintenance of remission was higher in the combination group (70.5%) than in the non-EN group (53.8%) [185]. A lack of pediatric evidence in this field exists. Notably, a “dose-dependent” response association appears to exist between the use of MEN and disease relapse [186]. A recent review performed by Gkikas et al. showed that consumption of >35% MEN was associated with significantly lower relapse rates compared to the control group, in 8 out of 10 (80%) studies (performed both in children and in adults). Conversely, in all four studies using ≤35% MEN, there was no additional benefit of MEN use in reducing clinical relapse [186]. Arguably, one of the main barriers to MEN consumption is low compliance, mainly due to formula palatability and patient’s exhaustion. Interestingly, compliance was not assessed in many of the studies in which MEN was not effective [180,181]. Therefore, treatment failure could have been biased by low acceptability of the dietary regimen rather than inadequate efficacy of MEN. The heterogeneity among different studies and the lack of RCTs studies in children remain some of the major limitations in this field of knowledge.

### 5.3. Food-Based Therapies in CD

EEN has shown high efficacy in the short term in inducing clinical remission. However, one of its main barriers is represented by monotony of food and taste fatigue experienced by patients. This is one of the major limitations to the wider access and availability of this type of therapy. Several food reintroduction protocols have been attempted after induction of remission using nutritional therapies [187,188,189]. Among the latter, single-food reintroduction with exclusion of symptom-aggravating foods was the most commonly used protocol [187], which was associated with lower clinical relapse rates, assessed by disease activity indices, compared to patients following an unrestricted diet. Subsequently, Woolner et al. [188] demonstrated that a low-fat, low-fiber diet (LOFFLEX) was associated with similar remission rates when compared to a symptom-alleviating, exclusion diet, arguing a higher acceptability for the patient. The results of these studies, conducted in an era in which markers of mucosal inflammation (endoscopies or FC) were not commonly used, need to be cautiously interpreted, as it remains questionable whether gut inflammation parallels symptoms. Recently, Logan et al. [182] demonstrated a rapid re-exacerbation of inflammation, demonstrated by a rise in FC, during the first 2–3 weeks after the reintroduction of whole food. Hence, re-establishment of a normal diet is associated with reactivation of intestinal inflammation, with MEN showing only a modest effect for the earliest weeks [182].

From this perspective, an understanding of how remission can be maintained through manipulation of diet represents a compelling unmet need. Therefore, it is no surprise that, in the past few years, multiple research groups have attempted to mimic EEN results with nutritional therapeutic strategies allowing access to whole food (Table 2.)

#### 5.3.1. Crohn’s Disease Exclusion Diet (CDED)

Among the various food-based therapies, Crohn’s Disease Exclusion Diet (CDED) currently represents the most clinically documented for the treatment of active CD. The pathophysiologic paradigm behind CDED is represented by the “bacterial penetration cycle hypothesis”, according to which the exposure of intestinal mucosa to adherent or penetrating bacterial species may act as a trigger for the immune system, thus generating inflammation, further barrier breakdown and a subsequent increase in the migration of harmful bacteria through a vicious cycle [190]. CDED was developed to exclude potential harmful foods that have been associated with alterations in host mucous layer [191], dysbiosis [192] and with the development of virulence factors [193] that may allow bacteria to attach to mucosa or to translocate the epithelial barrier. However, CDED is not all about exclusion, as this treatment aims to provide a consistent amount of high-quality protein and to deliver sugars mainly through foods rich in complex carbohydrates. Following these two principles of exclusion and inclusion, natural foods are combined with a variable volume of polymeric formula to meet the nutritional needs and to provide additional amounts of protein, calcium and vitamin D. The CDED nutritional regimen consists of different phases with incremental varieties of food allowed. Phase 1 lasts 6 weeks, aims to induce clinical remission and is the most restrictive one. Besides the elimination of dietary components that trigger inflammation, also fruit and vegetable consumption is restrained during the first phase in order to reduce fiber exposure. PEN is added to provide 50% of the nutritional needs. During phase 2, which lasts a further 6 weeks, access to food is broadened. To increase the flexibility of the diet and to improve adherence, fiber intake is increased so that almost all fruit and vegetables are allowed during this phase. Moreover, also potentially noxious foods (i.e., gluten, red meat, etc.), whose consumption is not permitted during phase 1, are gradually reintroduced in a controlled way. PEN consumption is reduced from 50% to 25% of the nutritional requirements [194]. Efficacy of CDED was first assessed in a cohort of 47 patients (34 children and 13 adults, mean age: 16 ± 5.6 years) [195]. Sigall-Boneh et al. [195] reported that such a dietary intervention, based on CDED combined with PEN at 50%, was able to induce clinical responses in nearly 80% and clinical remission in up to 70% of patients with mild-to-moderate CD. They also demonstrated the capacity of such a dietary regimen to normalize CRP in 70% of patients who achieved clinical remission. The abovementioned results were subsequently confirmed in a 12-week prospective RCT [34]. Seventy-eight children with mild-to-moderate newly diagnosed CD were randomly assigned to a group (*n* = 40) that received CDED plus PEN at 50% for 6 weeks followed by CDED with PEN at 25% from week 7 to 12 or a group (*n* = 38) that received EEN for the first 6 weeks and then PEN at 25% with a free diet [34]. The authors observed no differences in corticosteroid-free remission rates at week 6 (75% in CDED group vs. 59% in EEN group, *p* = 0.38). At week 12, the combination of CDED plus PEN was associated with higher remission rates than EEN (75.6% vs. 45.1%, *p* = 0.01). Additionally, the authors reported that CDED plus PEN was better tolerated than EEN (tolerance rates: 97.5% vs. 73.6%, *p* = 0.002). Furthermore, in a subsequent analysis of the abovementioned RCT, it was demonstrated that CDED + PEN was able to induce a rapid clinical response (at week 3), thus possibly identifying predictors of being in clinical remission at week 6 [196].

The efficacy of CDED was also demonstrated in another, unfortunately common, clinical scenario: the loss of response to biologic agents [197,198]. Sigall-Boneh et al. [199] reported the outcomes of 21 patients (11 children and 10 adults) with CD who had experienced loss of response (LoR) to a biologic agent despite dose escalation or combination therapy and received a course of 12 weeks of CDED plus PEN at 50%. At week 6, clinical remission was obtained in 13/21 patients (61.9%). Mean CRP levels decreased from 2.8 ± 3.4 mg/dL to 0.7 ± 0.5 mg/dL (*p* = 0.005) and mean albumin increased from 3.5 ± 0.6 g/dL to 3.8 ± 0.5 g/dL (*p* = 0.06). After the first two phases lasting 12 weeks, the maintenance phase begins. Occasional and controlled access to some type of foods not allowed during the earliest phases is now permitted. The duration of this phase is not strictly determined as it is intended to become a stable modification through a healthier lifestyle [194].

#### 5.3.2. CD Treatment-with-Eating (CD-TREAT)

CD-TREAT is an individualized, food-based therapy, which aims to recreate as closely as possible the composition of EEN using ordinary (solid) food through the exclusion of certain dietary components (i.e., gluten, lactose) and the combination of others (macronutrients, vitamins, minerals and fiber) [35]. The objective of such a dietary strategy is to mimic EEN’s capacity to modulate the gut microbiota composition [35]. Such properties were first assessed by Svolos et al. [35] in a prospective RCT enrolling 25 healthy subjects, who were randomly allocated to receive either EEN or CD-TREAT for 7 days, each with a 2-week washout in between. The authors reported that the fecal microbiota along with fecal metabolome significantly changed in the same direction for the two dietary strategies, with several parallel changes in metabolites and species [35]. The effects observed in healthy subjects were subsequently replicated in gut inflammation animal models [35]. Lastly, CD-TREAT has been administered in a course of 8 weeks to five children with active CD in a pilot study, showing efficacy in inducing clinical remission and in reducing FC [35]. Although promising, these proof-of-concept data need to be confirmed in an ongoing, adequately powered RCT [200].

## 6. Conclusions

Nutrition and growth are peculiar features of pediatric age. When dealing with children and adolescents affected by CD, the comprehensive management of the disease must extend beyond tight control of inflammation and cannot refrain from enhancement of nutritional status. Indeed, results from the recent literature have shown that sarcopenia and undernutrition are associated with a poorer disease course, complications and to a lower response to pharmacological therapies, whose weaponry is expanding yet currently limited in pediatric populations. Moreover, clinicians caring for children and adolescents with CD need to deal with the developmental milestones, such as growth and puberty, distinctive of this delicate period and with the expected long-lasting course of the disease. From this perspective, targeting simultaneously the nutritional deficiencies, which commonly occur in children with CD, with the resolution of gut inflammation and the correction of intestinal dysbiosis may provide synergistic benefits while minimizing risks and allowing sparing of the pharmacological weaponry. Such an intriguing research line requires well-designed studies in order to unravel the full potential of nutritional and dietary therapies for CD. Hopefully, in the foreseeable future, integration of the “omics” datasets using a system biology-based approach will help to elucidate the complex bond between diet and host along with allowing the development of novel precision medicine applications [201].

## Figures and Tables

**Table 1:** Summary of the studies using EEN as nutritional therapy in children with CD.

First Author	Study Design	Population	Intervention (Duration)	Control Group	Key Findings
Morin [139]	R	4 CD with growth failure	Elemental formula (6 weeks)	N/A	- Clinical efficacy; - Weight and height restoration.
Sanderson [140]	P/RCT	8 pts with active CD	Elemental formula (6 weeks)	8 CD patients treated with CS	- Significant clinical and biochemical improvement compared to steroids; - Improved body weight and linear growth.
Cohen-Dolev [24]	P/O	60 patients with newly diagnosed CD	Any formula (6–8 weeks)	87 matched patients treated with CS	- EEN was superior to CS for inducing remission; - EEN trended to superiority for height z-score compared to CS.
Levine [142]	P/O	43 patients with newly diagnosed CD	Any formula (6–8 weeks)	114 patients treated with CS 29 with 5-ASA	- EEN and CS effective in normalizing CRP at week 12; - In mild-to-moderate CD, EEN was superior to CS in normalizing CRP.
Lee [143]	P/O	22 patients with active CD	Any formula (6–8 weeks)	52 patients treated with anti-TNF-α 16 pts with PEN + unrestricted diet	- Clinical remission rates of EEN and anti-TNF-α were 88% and 84%, respectively, superior to those of PEN (64%); - EEN and anti-TNF-α but not PEN were effective in normalizing FC.
Grover [145]	P/O	26 patients with active CD	Any formula (6 weeks)	N/A	- 58% of patients achieved at least partial MH; - 21% of pts had complete transmural remission of ileal CD.
Borrelli [146]	P/RCT	19 patients with active CD	Polymeric formula (10 weeks)	18 patients treated with CS	- No differences were observed in clinical remission rates; - EEN was superior to CS in achieving MH.
Grover [147]	P/O	54 patients with active disease	Any formula (6 weeks)	N/A	- Achievement of complete MH (SES-CD) after EEN course was associated with a long-term sustained response.
Rubio [148]	R	45 patients with CD who received oral EEN	Polymeric formula (8 weeks)	61 patients treated with continuous EEN	- Similar remission rates; - Similar changes in PCDAI and inflammatory markers; - Higher weight gain in the continuous EEN group.
Buchanan [149]	R	110 patients with CD	Polymeric/ Elemental formula (8 weeks)	N/A	- Patients with terminal ileum disease (*n* = 4) had lower remission rates but no other difference was observed among various disease locations.
Afzal [150]	R	65 patients with active CD	Polymeric formula (8 weeks)	N/A	- Patients with colonic disease phenotype had significantly lower remission rates compared to ileal and ileocolonic localizations.
Belli [152]	P	8 patients with active CD and growth failure	Polymeric formula (intermittent administration over 1 year)	4 matched CD patients not treated with EEN	- Intermittent EEN administration showed significant decrease in CDAI and in CS use; - Intermittent EEN showed significant height and weight improvement

P: prospective; R: retrospective; O: observational; RCT: randomized controlled trial.

**Table 2:** Summary of the studies using food-based therapies in children with CD.

First Author	Study Design	Population	Intervention (Duration)	Control Group	Key Findings
Sigall-Boneh [183]	R	47 children and young adult pts with active CD	CDED + PEN (12 weeks, *n* = 40) CDED weeks, *n* = 7)	N/A	Clinical remission achieved in 24/34 children and 9/13 adults at wk 6 and maintained in 27/33 patients at week 12; Significant fall in clinical disease activity and inflammatory markers.
Sigall-Boneh [187]	R	21 children and young adult pts with treatment-refractory CD	CDED + PEN (12 weeks, *n* = 12) CDED (12 weeks, *n* = 4) Mod. EEN + CDED (2 + 12 weeks, *n* = 5)	N/A	13/21 pts refractory to biologic treatment achieved clinical remission; 9/17 of patients failing double biologic therapy achieved clinical remission; Significant decrease in serum markers of inflammation.
Levine [32]	P/RCT	40 pts with mild-to-moderate CD	CDED + PEN (12 weeks)	34 pts with mild-to-moderate CD treated with EEN	CDED+PEN was equally as effective as EEN in inducing remission at week 6; CDED+PEN was superior to EEN in maintaining remission at week 12; CDED+PEN was able to induce rapid remission (3 weeks);
Svolos [33]	OL	5 pts with active CD (PCDAI ≥ 12.5)	CD-TREAT (8 weeks)	N/A	CD-TREAT was able to induce clinical response in 80% and remission in 60% of patients; 80% of pts showed decrease in fecal calprotectin

P: prospective; R: retrospective; O: observational; RCT: randomized controlled trial.

## Data Availability

Not applicable.
